# Characterizing Perceived Control Over Daily Stress: Longitudinal Changes and Associations With Affect

**DOI:** 10.1093/geroni/igab046.826

**Published:** 2021-12-17

**Authors:** Eric Cerino, Jacqueline Mogle, Robert Stawski, Jonathan Rush, David Almeida

**Affiliations:** 1 Northern Arizona University, Flagstaff, Arizona, United States; 2 Penn State University, University Park, Pennsylvania, United States; 3 Oregon State University, Corvallis, Oregon, United States; 4 University of Victoria, Victoria, British Columbia, Canada; 5 Pennsylvania State University, University Park, Pennsylvania, United States

## Abstract

Perceived control is an important psychosocial correlate of healthy aging. Using data from the National Study of Daily Experiences (N=1,047, M=55.82 years, SD=10.35, 57.27% Female), we examined cross-sectional age-related differences and longitudinal aging-related change in perceived control over daily stress across 10 years and explored the influence of stressor control on negative affect (NA) and positive affect (PA). Stressor control, NA, and PA were obtained from telephone interviews over 8 consecutive days in measurement bursts conducted in ~2008 and ~2017. Longitudinal analyses revealed significant declines in stressor control across 10 years (p<.001). Cross-sectional analyses revealed marginally lower stressor control among older individuals (p<.10). Within-person associations revealed lower NA and higher PA on days when stressor control was higher than usual (ps<.001). Results suggest that stressor control declines with age and holds promise as an important component of daily stress processes with relevance for health and well-being outcomes across the lifespan.

